# Supplementary material for: Evidence of Zika virus circulation in asymptomatic pregnant women in Northeast, Brazil
Source: PLoS Negl Trop Dis. 2021 Jun 10;15(6):e0009412. doi: 10.1371/journal.pntd.0009412 (PMC8219130; doi:10.1371/journal.pntd.0009412)
Supplement: S1 Table — (DOCX) [file pntd.0009412.s001.docx]

Table S1**. Primers used in RT-qPCR for ZIKV detection (Y = T or C, R = A or G).**

| **Type of oligonucleotide (sense)** | **Sequence sense (5′– 3′)** | **Nucleotide position** |
| --- | --- | --- |
| **Primer Zika_qRT_F (+)** | AARTACACATACCARAACAAAGTG GT | 9271–9297 |
| **Primer Zika_qRT_R (-)** | TCCRCTCCCYCTYTGGTCTTG | 9352–9373 |
| **Probe Zika_qRT_P** | FAM-CTYAGACCAGCTGAAR-BBQ | 9304–9320 |

Source: Faye et al. (2013)^17^.
