# Supplementary material for: Evidence of Zika virus circulation in asymptomatic pregnant women in Northeast, Brazil
Source: PLoS Negl Trop Dis. 2021 Jun 10;15(6):e0009412. doi: 10.1371/journal.pntd.0009412 (PMC8219130; doi:10.1371/journal.pntd.0009412)
Supplement: Attachment — Submitted filename: Abstract Brazil asymptomatic women.docx [file pntd.0009412.s002.docx]

**I-Abstract** :

*-Method* : Sao Luis not mentioned in the method . You wrote “two maternity clinics (Benedito Leite and Maternity and Infant Care Unit of the University Hospital of the Federal University of Maranhão)” ; is it in Sao Luis ?

*-Results* : “The presence of ZIKV in umbilical cord blood was not related to morphological and/or psychomotor changes in the newborns” : for me, with this kind of conclusion, you cannot add “The circulation of ZIKV in asymptomatic pregnant women is important to improve prenatal care” . Not easy to see the importance of prenatal care if there are no morphological / psychomotor changes in the newborns ….We don’t see the urgency to improve prenatal care if there are no consequences.

**II- Article:**

**Background :**

- important to mention the outbreak in French Polynesia just before the one in Brazil (2014/2015) because, retrospectively,, they detected more foetal and newborn anomalies as well.
- within the **SUS** ( ?) in São Luís : what is a SUS
- state of Maranhão (MA) : where is it located in Brazil ?

**Method :**

The objectives of the study clearly articulated with a clear testable hypothesis stated. The study design is appropriate to address the stated objectives. The population is clearly described and appropriate for the hypothesis being tested . In the “Study type and area” section : you don t mention it is in Sao Luis city. Important to mention.

There is a correct statistical analysis used to support conclusions. the sample size (196) is sufficient to ensure adequate power to address the hypothesis being tested : however, the sample could be even bigger for more powerful conclusions. Unfortunate that the sample was of convenience.

There are correct statistical analysis used to support conclusions.

There are no concerns about ethical or regulatory requirements being met.

**Results**

The analysis presented match the analysis plan. The results are clearly and completely presented. One inconsistency in the number of pregnant women : 197/196- Check in the rapid test section. You should maybe remind the reader about IgM and IgG for Zika, time of persistence/time of appearence etc ... to understand and interpret better the results. Same p28, you don t give the percentage or results for the head size at birth despite the fact that you have table 6 and 7 showing some results, Use them +++

Regarding the figures, they are of sufficient quality for clarity. You mention two “table 3” and you have no “table 4”p 20.

**Discussion**

Not sure this sentence “Professionals should not handle teenage pregnancy (women 15–19 years of age), in the same way as adult pregnancy because teenagers are still undergoing growth and biopsychosocial development” is useful. It does not bring anything to your study.

Regarding sociodemographic factor : you just discuss the age and education but not the religion : do you want to elaborate a bit on this ?

For this sentence : “Regarding education, 67% of the pregnant women in the present study, had between 10 and 15 years of schooling and, therefore, a good level of education, which may **promote the beginning of prenatal care**”. Maybe you can replace by “**may suggest** **optimal/good prenatal care**” or **“may lead to the presumption of optimal/good prenatal care”.**

You mention that Chikungunya was tested but what about dengue ? what is your conclusion ? Please elaborate on this as this is simply described but not discussed.

**Conclusion**

Some of the are conclusions supported by the data presented but not all. Need to be much more elaborated. The limitations of analysis are clearly described maybe the 3 limitations “low complexity”, “diversity of the population” and “ low prevalence of ZIKV” can be elaborated a bit more. The authors discuss partially how these data can be helpful to advance our understanding of the topic under study. For me, the public health relevance can be addressed but need more in depth explanation on how this study can be translated into public health measures and real progress for prenatal care.

- Elaborate a bit more. Too short .
- You write “The study results showed the presence of ZIKV in the placenta and umbilical cord of women without clinical manifestations” : is it the first time it is demonstrated/shown ? If it is the first time, insist on it ++++
- I don t understand why your study shoes that ZIKV testing in prenatal care is crucial : if you demonstrate at the end that there is some virus in the placenta and in the umbilical cord but no consequences and no congenital malformation, what will be the objective ? the adding value ? What will bring the early testing for the decision of the mother to keep going on with the maternity ? What could be the consequences of damaged placenta : if there are please explain and stress the importance of prenatal care. You need to explain what prenatal care could prevent or help with your findings.
- Be careful with your English : “It is important (no “e”) to includ**e** ZIKV testing in prenatal care”
