# Supplementary material for: Evidence of Zika virus circulation in asymptomatic pregnant women in Northeast, Brazil
Source: PLoS Negl Trop Dis. 2021 Jun 10;15(6):e0009412. doi: 10.1371/journal.pntd.0009412 (PMC8219130; doi:10.1371/journal.pntd.0009412)
Supplement: Attachment — Submitted filename: Response to reviewers Zika.docx [file pntd.0009412.s003.docx]

Editor Comments

Overall
The present manuscript will be strengthened by a grammar review with a native speaker.

**Answer:** The English version was updated

Please, follow the PLoS Neglected Tropical Diseases guidelines.

**Answer:** The manuscript was updated.

Introduction

Please, edit the sentence:

“This is the first study of ZIKV infection diagnosis in asymptomatic pregnant women in the state of Maranhão, Brazil, using different methods.”

**Answer:** The sentence was updated to this study investigated the prevalence of Zika infection in asymptomatic pregnant women in Maranhão state, Northeast Brazil.

Methods

Please, inform the correct study design. For me, this was a cross-sectional study.

**Answer:** We included the study design. This is a cross-sectional study.

What the authors meant by: “Cases in which the tests could not be performed due to technical difficulties”. Please, clarify that sentence.

**Answer:** We included the study design. This is a cross-sectional study.

Provide a reference for the definition of microcephaly. Did the authors use INTERGROWTH 21st? If not used, this should be placed as a limitation of the study.

**Answer:** We used Intergrowth 21^st^ to define microcephaly.

Provide more details about the rapid test used. Was a rapid diagnostic test based on antigen detection? Or was a rapid diagnostic test based on host antibody detection? What is the sensitivity and specificity of the test? Was it used as a screening test (test-to-test)? Or was it used for diagnosis? This must be taken into account.

**Answer:** We included the study design. This is a cross-sectional study.

The authors must include a section on the definition they used for ZIKV infection. Was ZIKV infection considered a positive result for any of the tests? Depending on the sensitivities and specificities of the tests, this may have led to an important bias.

**Answer**: ZIKV infection was defined by laboratorial results (positivity of rapid test, ELISA and PCR) and exclusion of other arboviruses during the period of the study. This immunochromatographic test is double-path platform and evaluates presence of IgM and IgG antibodies with sensitivity and specificity around 95%. The test is negative for IgM and IgG for values of 0–22, indeterminate for values of 23–27, and positive for values equal to or higher than 28.

Data analysis. Authors should consider providing more details about data analysis, as well as including a multivariate analysis.

**Answer**: A digital databank was constructed using IBM SPSS Statistics for Windows, version 22.0. Data were transferred to a computerized databank using Epi-Info software for further analysis. A significance level of p < 0.05 and confidence interval of 95% were used. Variables with normal distributions were analyzed using parametric tests, while variables without normal distribution were analyzed using non-parametric tests. Qualitative variables were analyzed using chi-square and Fisher’s exact tests and odds ratios (ORs) (CI 95%). A multivariate analysis was performed using logistic regression model. Average, standard deviation, and minimum and maximum or median values were calculated for quantitative variables with normal distributions. The 25^th^ and 75^th^ percentiles were determined for variables with non-normal distributions. The association between epidemiological, clinical, and histopathological data and the laboratory results for ZIKV were evaluated by chi-square and Fisher’s exact tests, with p <0.05. All statistical analyses were performed using GraphPad Prism version 6 (GraphPad Software, CA, USA).

Results

The authors showed data on other arboviruses. But there are no details on how they ran the tests in the Methods section. The same occurs with the presentation of anatomopathological results.

**Answer**: We updated the results section, describing better arboviruses results and the presentation of anatomopathological results.

Conclusion

Please, leave the conclusion in a paragraph.

**Answer**: We updated the conclusion for one paragraph

**Methods**

Reviewer #1: The objectives of the study clearly articulated with a clear testable hypothesis stated. The study design is appropriate to address the stated objectives. The population is clearly described and appropriate for the hypothesis being tested and there is a correct statistical analysis used to support conclusions. the sample size (196) is sufficient to ensure adequate power to address the hypothesis being tested : however, the sample could be even bigger for more powerful conclusions. Unfortunate that the sample was of convenience.
There are correct statistical analysis used to support conclusions.
There are no concerns about ethical or regulatory requirements being met.

**Answer:** Thank you for the comments

Reviewer #2: The objective of the study mentioned in different sections of the manuscript is not the same. In the Background section it is stated that the aim of the study was to correlate ZIKV infection of the placenta and umbilical cord blood with teratogenicity, while in the Title the focus is on the evidence of Zika virus circulation in asymptomatic pregnant women and, finally, in the Methods it is on the prevalence of ZIKV infection in pregnant women. There should be a coherence between the different sections and the manuscript should be written accordingly.

**Answer:** We corrected the objectives in all the text.

Some aspects of the Methods need to be reviewed:
Sample size
Some points related to the sample size need to be clarified. How the expected frequency of ZIKV infection in the study population was taken into account ? What is meant by “number of diseases that caused microcephaly” and what is meant by “diagnosed cases causing fetal microcephaly” ?

**Answer:** The size of the study population was estimated based on the occurrence of ZIKV infection in the state of Maranhão during the first wave.

The number of participants recruited considered the prevalence of ZIKV infection that caused microcephaly diagnosed during prenatal care a reference hospital of São Luís, MA as shown in the supplementary Figure 1. A total of 203 pregnant was recruited, 7 were excluded because of difficulties in blood collection. A final sample of 196 pregnant women from the two maternities was then studied.

Infant Clinical Assessment
It is not informed which pediatric specialists (paediatricians? neuropediatricians ? other specialties?) performed the physical examination, if the same professionals examined all the children and if there was a standard form or medical record. These information are necessary to evaluate to which extent there was information bias and should be added to the Methods and further explored when discussing the strengths and limitations of the study in the Discussion section.

**Answer:** The infant clinical assessment was evaluated by paediatricians in the obstetric center.

Laboratory tests
The laboratory techniques were described in details. However there are some limitations in relation to the use of these techniques to diagnose Zika virus infection which may interfere in the interpretation of the results and therefore need to be added to the discussion. As the study population consisted of pregnant women who underwent vaginal birth or Cesarean section it seems that they were tested just in one point in time. How this fact and also the time window of positivity of these tests may have affected the findings of the study?

**Answer:** The aim of this study was to evaluate the presence of ZIKV in asymptomatic pregnant women. We performed different techniques (rapid test, ELISA, PCR and histopathological analyses in order to improve the diagnostic.

Maternal variables
The pregnant women were recruited when they were in active labor or undergoing elective Cesarean sections and reported to the emergency services of the maternities included in the study. Though the presence of symptoms compatible with Zika virus infection was not listed among the exclusion criteria we assume that they were all asymptomatic pregnant women because it is stated in the title of the manuscript. Were these symptoms investigated by the assistance team who performed the obstetric examination or by the research team ? A history of symptoms compatible with Zika virus infection during pregnancy was investigated? Were they asked about testing for Zika virus at some point during pregnancy

**Answer:** The symptoms were both investigated by assistance and research team.

**Results**

Reviewer #1: The analysis presented match the analysis plan. The results are clearly and completely presented. One inconsistency in the number of pregnant women : 197/196- Check in the rapid test section.You should maybe remind the reader about IgM and IgG for Zika, time of persistence/time of appearence etc ... to understand and interpret better the results. Same p28, you don t give the percentage or results for the head size at birth
Regarding the figures, they are of sufficient quality for clarity. You mention two “table 3” and you have no “table 4”p 20.

**Aswer:** We have included information about the tests (time of persistence, appearance, etc). We correct the number of patients (196) and described the percentage of results for the head size at birth

Reviewer #2: In relation to the results of the laboratory tests was there an overlapping of positivity between the PCR and IgM rsults?
It is not clear the rationale for testing the association between RT-qPCR results for placenta samples and evaluation of prenatal care quality.
Considering the small number of RT-PCR positivity in the umbilical cord and placenta is it worth to test the association with newborn variables ? Which would be the probability of detecting a difference if the difference exists, i.e., which would be the power of the test ?

**Aswer:** We have excluded the correlation between RT-PCR and prenatal care.

The rapid test, ELISA and RT-PCR were different and was not happened an overlapping among them.

**Conclusions**

Reviewer #1: Some of the are conclusions supported by the data presented but not all. Need to be much more elaborated. The limitations of analysis are clearly described maybe the 3 limitations “low complexity”, “diversity of the population” and “ low prevalence of ZIKV” can be elaborated a bit more. The authors discuss partially how these data can be helpful to advance our understanding of the topic under study. For me, the public health relevance can be addressed but need more in depth explanation on how this study can be translated into public health measures and real progress for prenatal care.

**Aswer:** We have improved the conclusions in order to better explain this.

Reviewer #2: The finding that ZIKV was circulating in the population of Maranhão in the first semester of 2018, as shown by the PCR results in the umbilical cord blood and placenta samples, is an important issue and should be further explored in the Discussion and Conclusions. Was there also evidence from the notification system that transmission was still happening? Are pregnant women with rash still being notified? If yes, are they being tested? Is there any evidence of circulation of ZIKV from other sources of information? What could be said about the transmission of ZIKV in Maranhao nowadays?
The discussion of the risks related to teenage pregnancy are out of the scope of the study and do not need to be included. Similarly, the comments made on the level of education of the participants are not linked to the ZIKV transmission. On the other hand, the implications that this finding of a relatively good level of education would have on the inference of the results of the study to pregnant women of the source population, or to the general population, are nor explored.
The comparison with the results of Brasil and colleagues (2016) could be enriched if the different epidemiological scenarios in which these studies were conducted is taken into account. Furthermore, it is worth mentioning if the diagnostic tools to identify adverse outcomes in both studies were comparable. There are other studies in the literature, in addition to the study of Noronha and colleagues (2016) and Shing and colleagues (2016), that could support the discussion of the frequency of adverse outcoms in children born to symptomatic and asymptomatic mothers.
In the limitations of the study it could be added the small sample size of women with laboratory evidence of ZIKAV infection during pregnancy which limits the power of the study to detect morphological and/or psychomotor changes in the newborns. In this way, could this lack of association be included in the conclusion of the study without other comments?
In the conclusion the authors say that It is important to include ZIKV testing in prenatal care. However they do not make clear on which finding this conclusion was based and for what reason this testing should be part of the prenatal care.

**Aswer:** We have improved the discussion, conclusion and strengths and limitations. We better explored the study of Brasil and col.

We have notified the Health Secretary about this study in order to prevent ZIKV and its consequences.

**Editorial and Data Presentation Modifications**

Reviewer #1: No editorial suggestion
Need to review the abstract as the conclusion is maybe not enough attractive.
Major modifications are needed in the discussion and conclusion to make this article much more relevant in term of Public Health.

**Answer:** We have improved the abstract, discussion and conclusion.

Reviewer #2: The manuscript could focus mainly on the finding of the laboratory evidence of the presence of ZIKV infection in asymptomatic pregnant women in this population and what it adds to the understanding of the circulation of ZIKV in the post-epidemic period.

**Answer:** We have corrected this

**Summary and General Comments**

Reviewer #1: This study seems quite innovative with important findings that need to be much more emphasized. More work is needed to link the finding with the practical implementation in prenatal care. What this study will change for pregnant women that might be asymptomatic with ZKV ? What could be the consequences ? What are the risks ? etc .... need more insights.
No concerns about dual publication, research ethics, or publication ethics.

**Aswer:** We have better described this in the manuscript, regarding the importance of ZIKV testing in prenatal care.

Reviewer #2: As mentioned in the previous items, the objective of the study should be stated clearly and the main objective should be the axis for the presentation and discussion of the results. The comparisons for which the authors did not present a rationale to support could be excluded and the interpretation of the results for which the sample size was too small should take into account this problem.
Follow a summary of the previous comments: a) Some points related to the sample size need to be clarified; b) more information is needed in relation to the evaluation of the children and its implications in interpreting the results; c) the limitations of the lab techniques to diagnose ZIKV infection, and their use in just one point in time, may affect the interpretation of the results need to be discussed; d) it is worth informing if a history of symptoms compatible with Zika virus infection during pregnancy was investigated and if these women reported to be tested for ZIKV infection previously; e) the conclusion needs to be rewritten and be based on the main findings.

**Answer:** We have corrected all items.
