# Supplementary material for: Evidence of Zika virus circulation in asymptomatic pregnant women in Northeast, Brazil
Source: PLoS Negl Trop Dis. 2021 Jun 10;15(6):e0009412. doi: 10.1371/journal.pntd.0009412 (PMC8219130; doi:10.1371/journal.pntd.0009412)
Supplement: Attachment — Submitted filename: Response to reviewers Zika.docx [file pntd.0009412.s004.docx]

Response to reviewers

We would like to thank you for your comments about this manuscript

In order to your requests, we have corrected some information in this study.

Sincerely,

Maria do Desterro Soares Brandão Nascimento

Reviewer's Responses to Questions

Reviewer #2: The authors say that the study investigated the prevalence of Zika infection in asymptomatic pregnant women in Maranhão state, Northeast Brazil. Though the statement is correct it may give the idea that the study was designed to estimate the prevalence of asymptomatic infection in pregnant women in the Maranhao State. In fact, these results could only be inferred to the two health units sampled in the study (internal validity). The extent to which it could be extrapolated to pregnant women in the Maranhão state depends on the similarity or differences between the sample (women studied), the target population (women attending the two maternities) and the population of pregnant women (external validity) in this state. Therefore it would be better to say “…study investigated the prevalence of Zika infection in asymptomatic pregnant women attending two public maternities in Maranhão state, Northeast Brazil’.

**Answer:** We have replaced the sentence to study investigated the prevalence of Zika infection in asymptomatic pregnant women attending two public maternities in Maranhão state, Northeast Brazil’.

The information in relation to the sample size calculation is still a bit confusing. As it is written in the text of the manuscript , there is an apparent inconsistency between the objective (This study investigated the prevalence of Zika infection in asymptomatic pregnant women …) and the parameter used to estimate the sample size (… the number of participants considered the prevalence of ZIKV infection that caused microcephaly). If the objective was not related to the pregnancy outcome (microcephaly) why was it considered in the sample size estimate? Please make clear in the text if the sample size was estimated based on the prevalence of ZIKV infection or on the prevalence of ZIKV infection that caused microcephaly. If the latter, please explain the reason. The questioning is not on the sample size that was achieved but on the parameters used to calculate it, and if they are adequate to the objective of the study

**Answer:** We clarified the information about sample sizer

Reviewer #2: The authors say in the discussion that it is not clear whether placental alterations are related to fetal adverse or neonatal outcomes or if they are associated with maternal–fetal transmission of ZIKV. However they do not recommend any action (systematic review ? meta-analysis ? any other type of pooled analysis of different studies) to elucidate this point. 
On the other hand they recommend to include ZIKV testing in prenatal care in order to better evaluate the newborns. This recommendation is not backed by the findings of the study. Introducing ZIKV testing to prenatal care is very complex and has several implications in relation to the follow-up of the mothers, in relation to the women’s decision to interrupt (or not) the pregnancy, in relation to the conditions that would be offered to the follow-up of the children, etc). Thus it would be better to withdraw this recommendation or discuss it in depth.  
In relation to the lack of association between ZIKV in umbilical cord blood and morphological and/or psychomotor abnormalities in the newborns, as it may be due to the low power of the study (as mentioned in the discussion), this possible explanation needs also to be clear also in the conclusion not to be misunderstood by the readers.

**Answer:** We included a statement about placental alterations**.** We removed this paragraph from conclusion

In the third paragraph of the Discussion the positivity for ZIKV infection of of the study of Brasil et al. (2016) are presented but they are not being used to interpret the results of this paper. In the previous version they were compared to the author’s findings but the reason why they differed was not explored. If a link with the author’s results is not made, it is not clear the reason to present them in the discussion.

**Answer:** We included a statement about this manuscript of Brasil and col.

Reviewer #2: I think the article has improved compared to the first version, but some points can be further worked on according to the recommendations made.

**Answer:** Thank you for your comments.
